# The Influence of Action Video Games on Attentional Functions Across Visual and Auditory Modalities

**DOI:** 10.3389/fpsyg.2021.611778

**Published:** 2021-06-02

**Authors:** Xia Wu, Ying Jiang, Yunpeng Jiang, Guodong Chen, Ying Chen, Xuejun Bai

**Affiliations:** ^1^Key Research Base of Humanities and Social Sciences of the Ministry of Education, Academy of Psychology and Behavior, Tianjin Normal University, Tianjin, China; ^2^Faculty of Psychology, Tianjin Normal University, Tianjin, China; ^3^Tianjin Social Science Laboratory of Students’ Mental Development and Learning, Tianjin, China; ^4^Institute of Psychology, Chinese Academy of Sciences, Beijing, China; ^5^Zhonghuan Information College, Tianjin University of Technology, Tianjin, China; ^6^Department of Social Psychology, Zhou Enlai School of Government, Nankai University, Tianjin, China

**Keywords:** action video games, visual and auditory modalities, attention network test, executive control, attentional functions

## Abstract

Attention can help an individual efficiently find a specific target among multiple distractors and is proposed to consist of three functions: alerting, orienting, and executive control. Action video games (AVGs) have been shown to enhance attention. However, whether AVG can affect the attentional functions across different modalities remains to be determined. In the present study, a group of action video game players (AVGPs) and a group of non-action video game players (NAVGPs) selected by a video game usage questionnaire successively participated in two tasks, including an attention network task-visual version (ANT-V) and an attention network task-auditory version (ANT-A). The results indicated that AVGPs showed an advantage in orienting under the effects of conflicting stimuli (executive control) in both tasks, and NAVGPs may have a reduced ability to disengage when conflict occurs in visual task, suggesting that the AVGs can improve guidance toward targets and inhibition of distractors with the function of executive control. AVGPs also showed more correlations among attentional functions. Importantly, the alerting functions of AVGPs in visual and auditory tasks were significantly related, indicating that the experience of AVGs could help us to generate a supramodal alerting effect across visual and auditory modalities.

## Introduction

Although some evidence has proven that playing violent video games is related to aggressive behaviors (Anderson et al., 2010; Prescott et al., 2018), the benefits of action video games (AVGs) on attention are one of the emerging fields for understanding their impact on child development (Granic et al., 2014), and researchers have further suggested that AVGs can influence mental health and education (Li and Tsai, 2013; Bavelier and Green, 2019). Attention refers to the process of selecting task-relevant stimuli and inhibiting task-irrelevant distractors, and it helps us to allocate the mental resources involved in a vast number of simultaneous inputs from visual, auditory and other sensory modalities. Relative to non-action video game players (NAVGPs), action video game players (AVGPs) are considered to have more attentional resources (Feng et al., 2007) and a better ability to accomplish selective attention in the visual modality (Green and Bavelier, 2003; Feng et al., 2007; Dye et al., 2009; Xiang and Hu, 2010; Hubert-Wallander et al., 2011), as well as greater phonological decoding speed in the auditory modality (Sandro and Sara, 2018). Although the experience of AVGs can improve probabilistic inference in both visual and auditory tasks (Green et al., 2010), whether it can influence attention across modalities remains to be revealed.

Attention is postulated to comprise a set of attentional functions, including an alerting function responsible for maintaining the state of response readiness to external warning stimuli, an orienting function responsible for selecting relevant information from numerous sensory inputs, and an executive control function responsible for detecting and resolving conflict among competing mental processes (Fan et al., 2002). Studies have found that AVGs can affect attentional functions. In terms of alerting, Donohue et al. (2010) found AVGPs to be good at determining the temporal sequence of multisensory stimuli. In terms of orienting, Hubert-Wallander et al. (2011) showed that AVGs could enhance the efficiency of selective visual attention. Franceschini et al. (2017) found that AVGs could improve the shifting of attention between visual and auditory modalities. In terms of executive control, Bavelier et al. (2012) and Krishnan et al. (2013) reported that AVGPs were better able to exclude task-irrelevant information. Bailey et al. (2010) and West et al. (2020) found that AVGs experience and training can reduce proactive cognitive control. However, the influence of AVGs on the interactions among attentional functions remain unclear.

Characterizing the supramodal and modality-specific mechanisms of the attentional functions could clarify their hierarchical structure. Specifically, executive control at a higher hierarchical level is considered to be responsible for coordinating mental computations and integrating information across modalities and has been proven to be supramodal. In contrast, alerting and orienting at a lower level are responsible for encoding sensory information, so they have been found to be modality-specific (Spagna et al., 2015, 2020). After the experience of quickly recognizing targets (e.g., in shooter games), responding to opponents (e.g., in fighting games) or changing formations (e.g., in sports games), the visual-auditory correlations of AVGPs may be altered in the modality-specific mechanisms of alerting and orienting.

In the present study, we investigated the influence of AVGs on the mechanisms and interactions of different attentional functions across visual and auditory modalities. By employing an attention network test (ANT, Fan et al., 2002), we can examine the efficiency of alerting, orienting and executive control simultaneously. AVGPs and NAVGPs were asked to successively complete visual and auditory versions of the ANT (Fan et al., 2009; Spagna et al., 2015) in a single session so that we could measure the within-subject correlations between attentional functions across visual and auditory tasks. The effects of AVGs on attention can be revealed by comparing AVGPs and NAVGPs with respect to the defined effects. The supramodal or modality-specific mechanisms of attentional functions were examined by the correlations between the attentional effects in the visual and auditory modalities. We predicted that the function of executive control and the correlations between visual and auditory task performance would be enhanced in the AVGP group relative to the NAVGP group. In addition, a supramodal mechanism for each attentional function and their interactions was found in the AVGP group, indicating the influence of AVGs experience on the mechanism of attention.

## Materials and Methods

### Participants

Sixty-eight adult volunteers were chosen based on a Video Game Usage Questionnaire and participated in this study. After excluding nine participants due to their low average accuracies (<70%) in both experiments, the remaining 59 participants (31 males and 28 females; mean ± standard deviation of age: 19.92 ± 1.51 years, range 18–24 years) were divided into the AVGP group (21 males and 10 females; mean ± standard deviation of age: 20.07 ± 1.65 years) and the NAVGP group (10 males and 18 females; mean ± standard deviation of age: 19.75 ± 1.33 years). All participants were undergraduate students, and they were chosen in equal numbers from different grades and majors (arts, sciences, engineering). All participants were right-handed had normal or corrected-to-normal visual acuity and normal hearing. They signed informed consent before the experiment, and a certain remuneration was given to each after the experiment. They completed the visual (ANT-V) and auditory (ANT-A) versions of the ANT with a break of 10 min, and the order of the tasks was balanced among the participants. The study was approved by the Ethics Committee of Tianjin Normal University.

The Video Game Usage Questionnaire was adapted from the Video Game Questionnaire (University of California, Berkeley database) and combined with assessment of current domestic use of games. The video games were classified into two types: action video games (AVGs), including action games (ACT, such as Devil May Cry, God of War, etc., Q9), first-person shooter games (FPS, such as CSGO, CrossFire, PUBG, etc., Q10), fighting games (such as: Street Fighter, The King of Fighters, etc., Q11), MOBA games (such as DOTA, League of Legends, Glory of Kings, etc., Q14), sports games (such as NBA, FIFA, Pro Evolution Soccer, etc., Q16), and real-time strategy games (RTS, such as StarCraft, Warcraft, etc., Q18); Non-action video games (NAVGs) includes strategy games, card games, puzzle games, etc (all the remaining questions). Referring to the research by Donohue et al. (2010), an AVGP is defined as a player who has played an average of more than 8 h of any type of action video game per week in the past 6 months; a NAVGP is defined as a player who has not played video games or has just played card games or puzzle games in the past 6 months.

### Attention Network Task-Visual Version (ANT-V)

**FIGURE 1:**
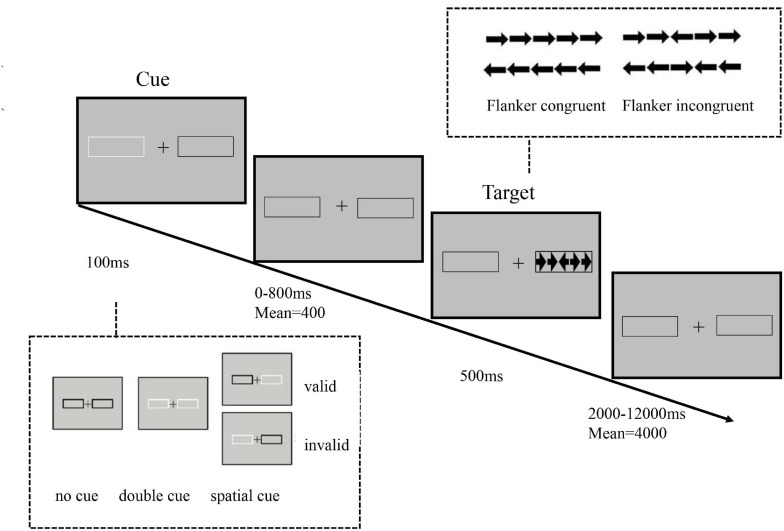
Schematic of the visual attention network task (ANT-V).

In the ANT-V (Figure 1), the stimuli were presented on a 15.6-inch monitor (1366 × 768 pixels, 60 Hz), and the participants were required to look at the central fixation cross (angle 1°) throughout the experiment. Participants were instructed to respond to the direction of the central arrow (left or right) as quickly and accurately as possible by pressing with the left or right index finger. The target arrow was flanked by four surrounding flanker arrows pointing in the same direction (congruent condition) or in the opposite direction (incongruent condition) with equal probability. The arrows (each visual angle was 0.58° with a gap of 0.06°) appeared in one of two black frames subtending 3.27° to the left and right of central fixation. In each trial, a cue occurred for 100 ms with a randomized duration (0, 400, or 800 ms) before the appearance of arrows, with three possible conditions: (1) no cue: the screen remained unchanged; (2) double cue: both black frames were briefly changed from black to white; (3) spatial cue: only one of the two black frames changed, with 75% of the cues in the same location as the target arrow (valid cues) and 25% of the cues in the opposite location (invalid cues). The durations of precue fixation and posttarget fixation were 3000 ms and a randomized value between 2,000 and 12,000 ms (with a mean of 4,000 ms), respectively. The task included four blocks, each with 72 trials, for a total of 288 trials. Of the total trial number, there were 48 trials for the no cue condition, 48 trials for the double cue condition, 48 trials for the invalid cue condition, and 144 for the valid cue condition. The entire experiment last approximately 40 min.

### Attention Network Task-Auditory Version (ANT-A)

**FIGURE 2:**
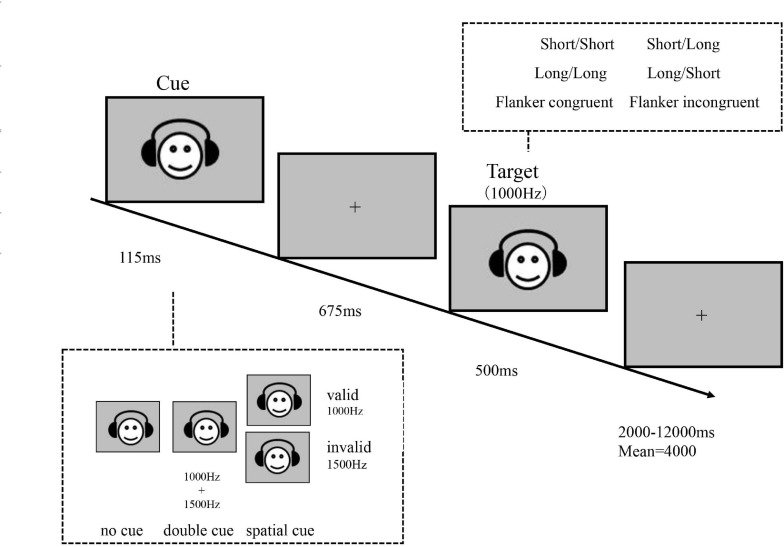
Schematic of the auditory attention network task (ANT-A).

In the ANT-A (Figure 2), the stimuli were presented on a 15.6-inch monitor (1366 × 768 pixels, 60 Hz), and the participants were required to look at the central fixation cross (angle 1°) and wear headphones (frequency response from 20 to 20,000 Hz) throughout the experiment. Participants were asked to indicate the duration (length) of a binaurally presented tone (short or long duration of either 30 or 150 ms, respectively) as quickly and accurately as possible by pressing the index button. The target tone was followed by another binaurally presented tone as a flanker, and the flanker tone was either the same length as the target (congruent condition) or the other length (incongruent condition) with equal probability. The tones occurred at either high (1,500 Hz) or low (1,000 Hz) frequency. In each trial, a cue occurred for 115 ms, with three possible conditions: (1) no cue: no tone prior to the target; (2) double cue: a mixed tone of 1,000 and 1,500 Hz occurred; (3) frequency cue: a 1,000-Hz or a 1,500-Hz tone occurred, with 75% of the cues at the same frequency as the target (valid cues) and 25% of the cues at the other frequency (invalid cues). Then a blank screen was presented for 675 ms before the onset of the target tone. The durations of precue fixation and posttarget fixation were 3,000 ms and a random value between 2,000 and 12,000 ms (with a mean of 4,000 ms), respectively. The operational definitions of the attentional effects and the experimental design were the same as those in the ANT-V.

### The Experimental Design and the Operational Definitions of the Attentional Effects

The factorial design was 2 (group: AVGP group, NAVGP group) × 4 (cue condition: no cue, double cue, valid cue, invalid cue) × 2 (target condition: congruent, incongruent). Error rate (ER) and reaction times (RTs) were calculated separately for each condition. RTs with incorrect answers or exceeding three standard deviations (SDs) of the mean of each condition were excluded from further analyses. The formulas used to define the attentional effects and interactions are listed in Table 1. All attentional effects and interactions were calculated to explore any possible effect of AVGs on attention. To investigate the effects of AVGs on attention, one-way ANCOVA was performed for the RTs and the error rate of each defined attentional effect and interaction with the group (AVGP and NAVGP) as the between-subject variable and gender as a covariate (following the methods of Spagna et al., 2015, 2018). A significant main effect of group would reveal a significant effect of AVGs. For each defined interaction with a significant effect, a further planned mixed ANCOVA with group (AVGP, NAVGP, between-subject variable) and the corresponding cue conditions (within-subject variables) was conducted to investigate the simple effects underlying this interaction. Moreover, to investigate the relationships among the attentional effects, Pearson’s correlation analyses were conducted for each attentional effect and interaction. FDR correction for multiple comparisons was employed. The *p*-values for each analysis were adjusted for multiple comparisons to a *P*_FDR_ < 0.05. Correlations were compared by using psychometrica.de^1^. SPSS software package (SPSS Inc. Chertsey, United Kingdom, version 22.0) was used to analyze the data.

**TABLE 1:** Operational definitions of the attentional effects and interactions.

	**Testing condition**	**Minus**	**Reference condition**
**Attentional effects**			
Alerting	No cue		Double cue
Disengaging	Invalid cue		Double cue
Orienting	Double cue		Valid cue
Validity	Invalid cue		Valid cue
Conflict	Incongruent cue		Congruent cue
**Interactions**			
Alerting by conflict	No cue, incongruent *minus* no cue, congruent		Double cue, incongruent *minus* double cue, congruent
Disengaging by conflict	Invalid cue, incongruent *minus* invalid cue, congruent		Double cue, incongruent *minus* double cue, congruent
Orienting by conflict	Double cue, incongruent *minus* double cue, congruent		Valid cue, incongruent *minus* valid cue, congruent
Validity by conflict	Invalid cue, incongruent *minus* invalid cue, congruent		Valid cue, incongruent *minus* valid cue, congruent

*The conflict effect reflects the function of executive control.*

## Results

### Differences in Attentional Effects Between AVGPs and NAVGPs in the ANT-V

**FIGURE 3:**
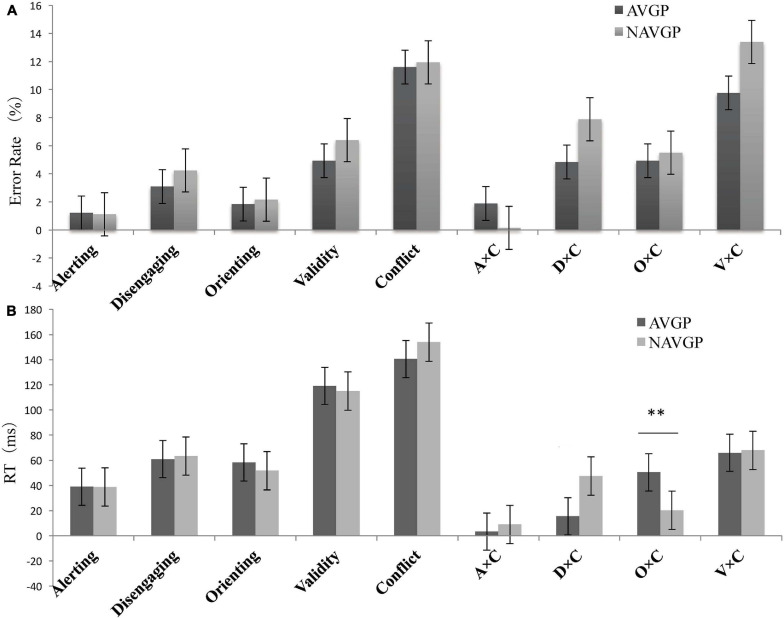
**(A)** Error rate (ER, %) and **(B)** Reaction times (RTs) as a function of the attentional effects of different groups in the ANT-V. Error bars represent the standard error of the mean. A × C, alerting by conflict; D × C, disengaging by conflict; O × C, orienting by conflict; V × C, validity by conflict. ^∗∗^*p* < 0.01.

The error rate and RTs for correct responses (*M* ± *SD*) of different groups for each condition and attentional effect are shown in Table 2 and Figure 3, respectively.

**TABLE 2:** Mean (SD) reaction time (RTs, ms) and error rate (ER, %) for each condition in the ANT-V.

**Group**		**Double cue**	**No cue**	**Valid cue**	**Invalid cue**
**RT**					
AVGP	Congruent	536 (53.00)	573 (50.28)	503 (52.95)	589 (53.68)
	Incongruent	684 (62.33)	725 (73.84)	601 (67.45)	753 (84.58)
NAVGP	Congruent	542 (55.08)	576 (65.65)	500 (51.37)	581 (58.16)
	Incongruent	687 (89.26)	730 (89.49)	625 (86.14)	774 (99.42)
**Error rate**					
AVGP	Congruent	0.40 (1.25)	0.67 (1.56)	1.03 (1.76)	1.08 (2.63)
	Incongruent	11.56 (12.06)	13.71 (13.23)	7.26 (7.88)	17.07 (13.83)
NAVGP	Congruent	0.15 (0.79)	1.19 (2.23)	0.74 (1.22)	0.45 (1.73)
	Incongruent	11.46 (12.71)	12.65 (16.14)	6.55 (8.96)	19.64 (19.51)

One-way ANCOVA was conducted for the error rate of each attentional effect and interaction. The results showed that two groups were not significantly different in the average performance in all conditions (the average performance on all conditions) [*F*(1,56) = 0.313, *p* = 0.578], alerting [*F*(1,56) = 0.003, *p* = 0.954], disengaging [*F*(1,56) = 0.526, *p* = 0.471], orienting [*F*(1,56) = 1.239, *p* = 0.270], validity [*F*(1,56) = 1.837, *p* = 0.181], conflict [*F*(1,56) = 0.554, *p* = 0.460], alerting by conflict [*F*(1,56) = 0.966, *p* = 0.330], disengaging by conflict [*F*(1,56) = 0.746, *p* = 0.391], orienting by conflict [*F*(1,56) = 1.232, *p* = 0.272], nor validity by conflict [*F*(1,56) = 2.350, *p* = 0.131].

One-way ANCOVA was conducted for the RTs of each attentional effect and interaction. The results showed that two groups were not significantly different in the average performance in all conditions [*F*(1,56) = 0.044, *p* = 0.835], alerting [*F*(1,56) = 0.034, *p* = 0.854], disengaging [*F*(1,56) = 0.014, *p* = 0.905], orienting [*F*(1,56) = 1.802, *p* = 0.185], validity [*F*(1,56) = 1.068, *p* = 0.306], conflict [*F*(1,56) = 1.379, *p* = 0.245], alerting by conflict [*F*(1,56) = 0.654, *p* = 0.422], nor validity by conflict [*F*(1,56) = 0.006, *p* = 0.941].

**FIGURE 4:**
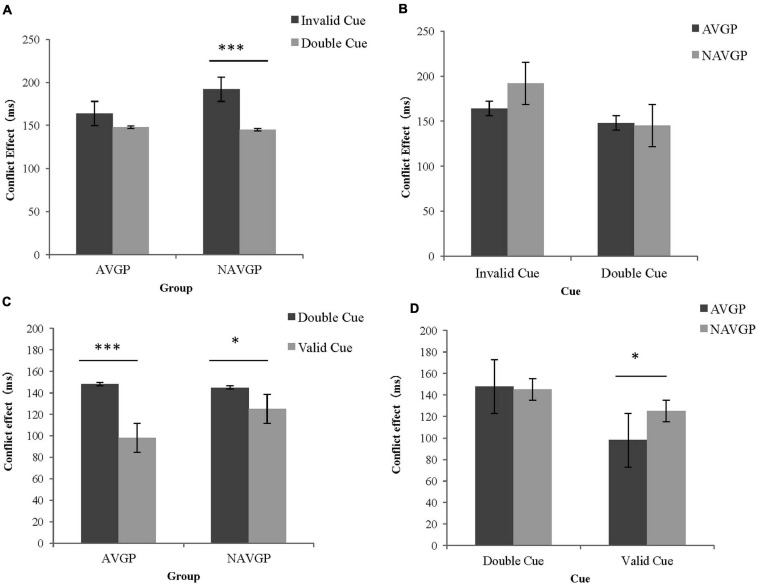
Conflict effects of different groups under different cue conditions in the ANT-V. **(A)** The conflict effects in AVGPs and NAVGPs under invalid cues and double cues; **(B)** The conflict effects in AVGPs and NAVGPs under invalid cues and double cues; **(C)** The conflict effects of AVGPs and NAVGPs under double cues and valid cues; **(D)** The conflict effects of AVGPs and NAVGPs under double cues and valid cues. Error bars represent the standard error of the mean. ^∗^*p* < 0.05, ^∗∗∗^*p* < 0.001.

However, a marginally significant difference in RTs between the two groups for disengaging by conflict [*F*(1,56) = 3.734, *p* = 0.058, *ω^2^* = 0.044], suggesting that the effects tend to be lower for AVGPs (15.52 ± 51.68) than for NAVGPs (47.49 ± 57.09)^2^, although the effect size was relatively small. To explore the separate effect on disengaging by conflict in different groups and the underlying simple effect in this interaction, a further mixed ANCOVA for the conflict effects with group (AVGP, NAVGP) and cue condition (invalid cue, double cue) was performed. The simple effect analysis of the interaction showed no significant difference in the reaction to conflict between two the cue conditions in AVGPs [*F*(1,56) = 2.820, *p* = 0.099], but the reaction to conflicting stimuli in the context of invalid cues was significantly lower than that with double cues in NAVGPs [*F*(1,56) = 18.702, *p* < 0.001] (Figure 4A), suggesting that NAVGPs may have reduced ability to disengage attention when conflict occurs. There was no significant difference in the conflict effect between the two groups, either for the invalid cue [*F*(1,56) = 2.475, *p* = 0.121] or the double cue [*F*(1,56) = 0.017, *p* = 0.898] (Figure 4B).

In addition, a significant difference in RTs between the two groups for orienting by conflict [*F*(1,56) = 7.081, *p* = 0.010, *ω^2^* = 0.094] showed that the effects were significantly higher for AVGPs (50.39 ± 40.21) than for NAVGPs (20.35 ± 41.09). To explore the separate effect on orienting in the context of conflicting stimuli in different groups and the underlying simple effect in this interaction, a further mixed ANCOVA for the conflict effects with group (AVGP, NAVGP) and cue condition (double cue, valid cue) was performed. The simple effect analysis of the interaction showed that RTs for conflicting stimuli in the context of double cues was significantly higher than that in the context of valid cues in both AVGPs [*F*(1,56) = 44.420, *p* < 0.001] and NAVGPs [*F*(1,56) = 6.517, *p* = 0.013] (Figure 4C), suggesting that both AVGPs and NAVGPs retained the ability to orient when conflict occurred. There was no significant difference in the conflict effect between the two groups in the double cue condition [*F*(1,56) = 0.017, *p* = 0.898], but the conflict effect was significantly higher for AVGPs than for NAVGPs in the valid cue condition [*F*(1,56) = 4.713, *p* = 0.034] (Figure 4D), indicating that AVGPs possess increased ability to resolve conflict with the valid orientation.

### The Correlations Between Attentional Effects for AVGPs and NAVGPs in the ANT-V

The coefficients of correlation between RTs to different attentional effects for AVGPs and NAVGPs are shown in Table 3. The AVGP group showed a significant positive correlation between disengaging and the interaction of validity and conflict (*r* = 0.655, *p* = 0.001) and a significant positive correlation between conflict and the interaction of validity and conflict (*r* = 0.521, *p* = 0.015). No such correlations were found for NAVGPs (*r* = 0.444, *p* = 0.052; *r* = 0.099, *p* = 0.757). NAVGPs showed a significant negative correlation between alerting and orienting (*r* = −0.662, *p* = 0.001), a significant positive correlation between alerting and conflict (*r* = 0.472, *p* = 0.037), disengaging and conflict (*r* = 0.471, *p* = 0.037), orienting and validity (*r* = 0.520, *p* = 0.026), orienting and the interaction of validity and conflict (*r* = 0.493, *p* = 0.035), and validity and the interaction of disengaging and conflict (*r* = 0.601, *p* = 0.005). However, no such correlations were found for AVGPs (*r* = −0.438, *p* = 0.052; *r* = 0.227, *p* = 0.340; *r* = 0.422, *p* = 0.058; *r* = 0.391, *p* = 0.088; *r* = 0.010, *p* = 0.980; *r* = 0.279, *p* = 0.231, respectively).

**TABLE 3:** Correlation coefficients for the attentional effects in different groups in the ANT-V.

**Group**		**Overall**	**Alerting**	**Disengaging**	**Orienting**	**Validity**	**Conflict**	**A × C**	**D × C**	**O × C**
AVGP	Alerting	0.190								
	Disengaging	0.431	0.474*							
	Orienting	–0.329	–0.438	–0.227						
	Validity	0.208	0.183	0.808***	0.391					
	Conflict	0.515*	0.227	0.422	–0.112	0.331				
	A × C	0.334	0.092	0.156	–0.152	0.056	0.209			
	D × C	0.340	0.271	0.439	–0.223	0.279	0.180	0.593**		
	O × C	–0.088	–0.005	0.134	0.298	0.306	0.324	−0.563**	−0.591**	
	V × C	0.327	0.322	0.655**	0.010	0.625**	0.521*	0.187	0.652**	0.226
NAVGP	Alerting	0.255								
	Disengaging	0.181	0.478*							
	Orienting	0.149	−0.662**	–0.239						
	Validity	0.268	–0.063	0.705***	0.520*					
	Conflict	0.630**	0.472*	0.471*	–0.235	0.243				
	A × C	–0.442	–0.142	0.125	–0.011	0.102	–0.076			
	D × C	–0.097	–0.174	0.408	0.331	0.601**	0.117	0.495*		
	O × C	0.196	–0.015	–0.003	0.165	0.118	–0.037	−0.586**	−0.475*	
	V × C	0.048	–0.202	0.444	0.493*	0.751***	0.099	0.081	0.721***	0.268

Overall, the average performance over all conditions; A × C, alerting by conflict; O × C, orienting by conflict; V × C, validity by conflict. *p < 0.05, **p < 0.01, ***p < 0.001.

The results and corresponding *p*-value from comparing the correlation coefficients of the attentional effects in AVGPs to those in NAVGPs are shown in Table 4. Relative to NAVGPs, AVGPs exhibited higher correlations between the average performance in all conditions and the interaction between alerting and conflict (*z* = 2.990, *p* = 0.001), alerting and the interaction of validity and conflict (*z* = 1.957, *p* = 0.025), and conflict and the interaction of validity and conflict (*z* = 1.740, *p* = 0.041). However, relative to AVGPs, NAVGPs showed higher correlations between the average performance in all conditions and orienting (*z* = −1.791, *p* = 0.037), orienting and the interaction of disengaging and conflict (*z* = −2.076, *p* = 0.019) and orienting and the interaction of validity and conflict (*z* = −1.926, *p* = 0.027). The results described above showed that, in the visual modality, the correlation between alerting and conflict was stronger for AVGPs, while the correlations between the functions of orienting (including the effects of validity, orienting, disengaging) and conflict were tighter for NAVGPs.

**TABLE 4:** Comparison of the correlation coefficients for the attentional effects in different groups in the ANT-V.

	**Overall**	**Alerting**	**Disengaging**	**Orienting**	**Validity**	**Conflict**	**A × C**	**D × C**	**O × C**
Alerting	–0.248								
Disengaging	1.011	–0.019							
Orienting	−1.791*	1.190	0.049						
Validity	–0.234	0.901	0.882	–0.591					
Conflict	–0.623	–1.023	–0.226	0.462	0.347				
A × C	2.990**	0.853	0.116	–0.518	–0.170	1.047			
D × C	1.639	1.646	0.136	−2.076*	–1.481	0.232	0.506		
O × C	–1.041	0.036	0.500	0.509	0.719	1.358	0.123	–0.591	
V × C	1.061	1.957*	1.115	−1.926*	–0.878	1.740*	0.394	–0.470	–0.163

Overall, the average performance over all conditions; A × C, alerting by conflict; O × C, orienting by conflict; V × C, validity by conflict. *p < 0.05, **p < 0.01.

### Differences in Attentional Effects Between AVGPs and NAVGPs in the ANT-A

**FIGURE 5:**
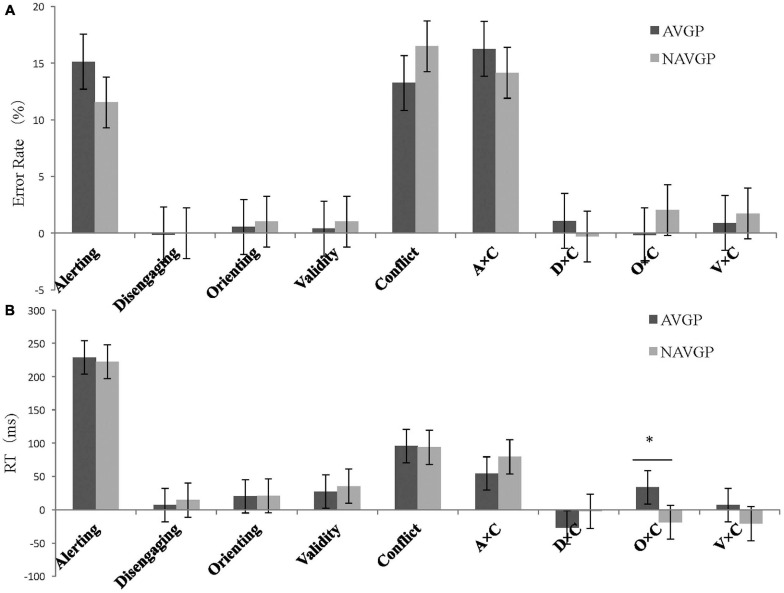
**(A)** Error rate (ER, %) and **(B)** Reaction time (RTs) as a function of the attentional effects for different groups in the ANT-A. Error bars represent the standard error of the mean. A × C, alerting by conflict; D × C, disengaging by conflict; O × C, orienting by conflict; V × C, validity by conflict. ^∗^*p* < 0.05.

Error rate and RTs for correct responses (*M* ± *SD*) of different groups for each condition and attentional effect are shown in Table 5 and Figure 5, respectively.

**TABLE 5:** Mean (SD) reaction time (RTs, ms) and error rate (ER, %) for each condition in the ANT-A.

**Group**		**Double cue**	**No cue**	**Valid cue**	**Invalid cue**
**RT**					
AVGP	Congruent	753 (119.29)	955 (123.86)	750 (117.17)	773 (106.34)
	Incongruent	850 (176.26)	1106 (162.67)	813 (150.72)	844 (160.25)
NAVGP	Congruent	756 (124.90)	939 (108.53)	726.(118.95)	772 (130.95)
	Incongruent	826 (171.93)	1089 (172.47)	815 (180.03)	840 (189.56)
**Error rate**					
AVGP	Congruent	6.45 (6.53)	13.44 (10.41)	5.82 (4.10)	5.78 (6.60)
	Incongruent	15.32 (12.14)	38.58 (18.63)	14.87 (9.50)	15.73 (13.55)
NAVGP	Congruent	5.21 (6.07)	9.67 (9.49)	5.21 (4.56)	5.36 (6.20)
	Incongruent	18.75 (12.65)	37.35 (13.86)	16.72 (9.74)	18.60 (16.06)

One-way ANCOVA was conducted for the error rate of each attentional effect and interaction. The results showed that two groups were not significantly different in the average performance in all conditions [*F*(1,56) = 0.021, *p* = 0.885], alerting [*F*(1,56) = 1.203, *p* = 0.277], disengaging [*F*(1,56) = 0.010, *p* = 0.921], orienting [*F*(1,56) = 0.027, *p* = 0.870], validity [*F*(1,56) = 0.057, *p* = 0.811], conflict [*F*(1,56) = 0.834, *p* = 0.365], alerting by conflict [*F*(1,56) = 0.670, *p* = 0.417], disengaging by conflict [*F*(1,56) = 0.180, *p* = 0.673], orienting by conflict [*F*(1,56) = 0.426, *p* = 0.517], nor validity by conflict [*F*(1,56) = 0.006, *p* = 0.939].

One-way ANCOVA was conducted for the RTs of each attentional effect and interaction. The results showed that two groups were not significantly different in the average performance in all conditions [*F*(1,56) = 0.149, *p* = 0.701], alerting [*F*(1,56) = 0.012, *p* = 0.912], disengaging [*F*(1,56) = 1.369, *p* = 0.247], orienting [*F*(1,56) = 0.037, *p* = 0.849], validity [*F*(1,56) = 1.404, *p* = 0.241], conflict [*F*(1,56) = 0.010, *p* = 0.919], alerting by conflict [*F*(1,56) = 0.519, *p* = 0.474], disengaging by conflict [*F*(1,56) = 1.219, *p* = 0.274], nor validity by conflict [*F*(1,56) = 0.889, *p* = 0.350].

**FIGURE 6:**
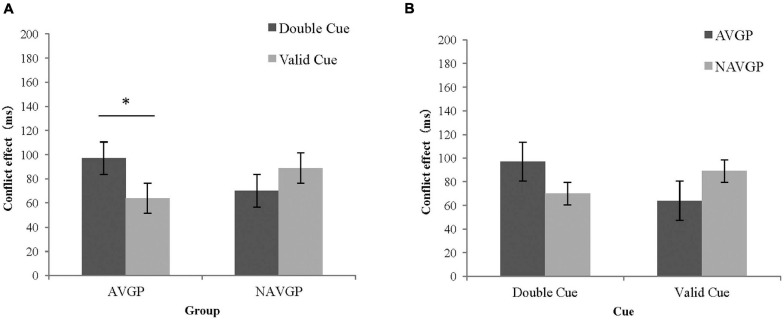
Conflict effects of different groups under different cue conditions in the ANT-A **(A)** The conflict effects in AVGPs and NAVGPs under double cues and valid cues; **(B)** The conflict effects in AVGPs and NAVGPs under double cues and valid cues. Error bars represent the standard error of the mean. ^∗^*p* < 0.05.

However, a significant difference in RTs between two groups was found in the interaction between orienting and conflict [*F*(1,56) = 6.466, *p* = 0.014, *ω^2^* = 0.084], showing that the effects were significantly higher for AVGPs (33.59 ± 69.21) than for NAVGPs (−18.85 ± 71.25). To explore the separate effects of orienting on conflict in different groups and the underlying simple effect in this interaction, a further mixed ANCOVA for the conflict effects with group (AVGP, NAVGP) and cue condition (double cue, valid cue) was performed. Further simple effect analysis of the interaction showed that the RTs for conflicting stimuli in context of double cues was significantly higher than that in the context of valid cues in AVGPs [*F*(1,56) = 6.094, *p* = 0.017], but no such difference was found for NAVGPs [*F*(1,56) = 1.580, *p* = 0.214] (Figure 6A), suggesting that AVGPs had increased ability to orient when conflict occurred. There were no significant differences in the conflict effect between the two groups in the context of double cues [*F*(1,56) = 0.936, *p* = 0.338] or in the context of valid cues [*F*(1,56) = 1.905, *p* = 0.173] (Figure 6B).

### The Correlations Between Attentional Effects in AVGPs and NAVGPs in the ANT-A

The coefficients of correlation in RTs for different attentional effects in AVGPs and NAVGPs are shown in Table 6. AVGPs showed a significant negative correlation between alerting and orienting (*r* = −0.481, *p* = 0.031), orienting and the interaction between disengaging and conflict (*r* = −0.485, *p* = 0.031), a significant positive correlation between disengaging and the interaction between disengaging and conflict (*r* = 0.475, *p* = 0.031), disengaging and orienting (*r* = 0.509, *p* = 0.022), and the interaction between alerting and conflict and the interaction between disengaging and conflict (*r* = 0.517, *p* = 0.022). No such correlations were found for NAVGPs (*r* = −0.078, *p* = 0.866; *r* = 0.163, *p* = 0.596; *r* = 0.281, *p* = 0.405; *r* = −0.469, *p* = 0.080; *r* = 0.266, *p* = 0.424, respectively).

**TABLE 6:** Correlation coefficients for the attentional effects in different groups in the ANT-A.

**Group**		**Overall**	**Alerting**	**Disengaging**	**Orienting**	**Validity**	**Conflict**	**A × C**	**D × C**	**O × C**
AVGP	Alerting	–0.160								
	Disengaging	–0.314	0.409							
	Orienting	0.294	−0.481*	−0.509*						
	Validity	–0.145	0.115	0.774***	0.151					
	Conflict	0.724***	–0.181	–0.142	0.285	0.046				
	A × C	–0.197	0.439	0.281	–0.326	0.083	–0.170			
	D × C	–0.028	0.303	0.475*	−0.485*	0.189	0.114	0.517*		
	O × C	0.313	–0.022	–0.194	0.341	0.027	0.289	−0.610**	−0.545*	
	V × C	0.225	0.338	0.397	–0.289	0.244	0.371	0.104	0.724***	0.184
NAVGP	Alerting	–0.246								
	Disengaging	0.223	0.451							
	Orienting	–0.098	–0.078	–0.469						
	Validity	0.191	0.461	0.839***	0.088					
	Conflict	0.784***	–0.453	–0.017	0.002	–0.017				
	A × C	0.306	0.173	0.246	–0.162	0.177	0.277			
	D × C	0.173	–0.128	0.281	0.163	0.417	0.030	0.266		
	O × C	–0.247	–0.020	–0.307	0.068	–0.304	–0.105	−0.631**	−0.563*	
	V × C	–0.033	–0.167	0.037	0.252	0.197	–0.064	–0.285	0.629**	0.289

Overall, the average performance over all conditions; A × C, alerting by conflict; O × C, orienting by conflict; V × C, validity by conflict. *p < 0.05, **p < 0.01, ***p < 0.001.

The results and corresponding *p*-value from comparisons of the correlation coefficients of the attentional effects in AVGPs to those in NAVGPs are shown in Table 7. Compared to NAVGPs, AVGPs exhibited higher correlations between average performance in all groups and interaction of orienting and conflict (*z* = 2.097, *p* = 0.018), between alerting and interaction of validity and conflict (*z* = 1.890, *p* = 0.029), and between orienting and interaction of disengaging and conflict (*z* = −2.523, *p* = 0.006). The results described above showed that, in the auditory modality, the correlations between attentional functions were stronger for AVGPs than for NAVGPs.

**TABLE 7:** Comparison of the correlation coefficients for the attentional effects in different groups in the ANT-A.

	**Overall**	**Alerting**	**Disengaging**	**Orienting**	**Validity**	**Conflict**	**A × C**	**D × C**	**O × C**
Alerting	0.328								
Disengaging	−2.008*	–0.190							
Orienting	1.457	–1.623	–0.193						
Validity	–1.235	–1.390	–0.675	0.231					
Conflict	–0.508	1.110	–0.460	1.057	0.232				
A × C	−1.872*	1.078	0.136	–0.636	–0.350	−1.661*			
D × C	–0.736	1.608	0.829	−2.523**	–0.919	0.308	1.087		
O × C	2.097*	–0.008	0.435	1.043	1.239	1.461	0.122	0.092	
V × C	0.950	1.890*	1.393	−2.017*	0.178	1.648	1.443	0.640	–0.405

Overall, the average performance over all conditions; A × C, alerting by conflict; O × C, orienting by conflict; V × C, validity by conflict. *p < 0.05, **p < 0.01.

### The Correlations Between RTs for Visual and Auditory Modalities for Attentional Effects in AVGPs and NAVGPs

The coefficients of correlation between RTs for ANT-V and ANT-A tasks for attentional effects in different groups are shown in Table 8. There were significant positive correlations between the RTs of the two tasks averaged over all conditions in AVGPs (*r* = 0.392, *p* = 0.029) and NAVGPs (*r* = 0.417, *p* = 0.027). Importantly, a significant positive correlation was observed between alerting in the two tasks in AVGPs (*r* = 0.396, *p* = 0.027), whereas there was no significant correlation in NAVGPs (*r* = −0.339, *p* = 0.078). Comparison of correlation coefficients for attentional effects in different modalities between AVGPs and NAVGPs showed that AVGPs had better ability to generate a supramodal alerting function (*z* = 2.805, *p* = 0.003).

**TABLE 8:** Correlation coefficients for the attentional effects in different groups between the ANT-V and the ANT-A.

	**Overall**	**Alerting**	**Disengaging**	**Orienting**	**Validity**	**Conflict**	**A × C**	**D × C**	**O × C**	**V × C**
AVGP	0.392*	0.396*	0.110	0.146	0.233	0.195	0.056	0.022	–0.036	0.138
NAVGP	0.417*	–0.339	0.157	0.237	–0.090	–0.051	–0.144	–0.117	0.135	–0.060
*z*	–0.109	2.805	–0.174	–0.344	1.191	0.903	0.731	0.507	–0.625	0.723
*p*	0.457	0.003	0.431	0.366	0.117	0.183	0.232	0.306	0.266	0.235

Overall, the average performance over all conditions; A × C, alerting by conflict; O × C, orienting by conflict; V × C, validity by conflict. *p < 0.05.

## Discussion

In the present study, in a within-subject design, we investigated the influence of action video games (AVGs) on the attentional functions across visual and auditory modalities by simultaneously testing the effects on alerting, orienting and executive control. Our results found that, relative to the results for non-action video game players (NAVGPs), action video game players (AVGPs) showed a significant interaction between orienting and conflict (executive control) functions in both visual and auditory modalities, suggesting that AVGPs have an advantage in orienting when conflict occurs. Furthermore, AVGPs also exhibited more correlations among the three attentional functions, indicating their benefits for binding attentional processing and for efficiency of target identification. Interestingly, a significant correlation between the performance of visual and auditory tasks was found in the alerting effect in AVGPs, indicating that a supramodal alerting function that operates independently of sensory modality can be influenced by the experience of AVGs.

The significant interaction in RTs between orienting and conflict in the AVGP group suggests that AVGs directly benefit orienting to executive control. The marginally significant interaction in RTs between disengagement and conflict in the NAVGP group may suggest that nAVGs may reduce the ability to disengage to executive control. When the conflicting stimuli occurs, AVGPs can quickly focus their attention on the target while NAVGPs may not be good at disengaging their attention from task-irrelevant distractors (Callejas et al., 2005). Consistent with the findings of Dye et al. (2009) and Gao et al. (2018), the present results indicate that the experience of AVG is associated with enhanced guidance of the target and inhibition of distractors. As frequent players of AVGs, AVGPs can rapidly allocate attention to the informative stimulus (orienting) and disengage attention from irrelevant stimuli (disengagement) and then employ the engagement of attentional resources to detect and resolve the conflict embedded in a complex game environment. It is noteworthy that the advantage from AVGs was found in both visual and auditory tasks, indicating a general common influence of AVGs on attentional functions, irrespective of visual or auditory modalities.

The significant correlation between RTs for visual and auditory tasks in the alerting function for AVGPs indicates that a supramodal, irrespective of specific modality, alerting effect can be influenced by AVGs. Inconsistent with results of the present study, by examining the ordinary people, Spagna et al. (2015) found that alerting relies on modality-specific processes, which can be explained in that the alerting effect requires no active reaction in the auditory modality but requires attention for turning toward targets in the visual modality (Posner, 1978). However, after the experience of playing AVGs, the modality-specific alerting function becomes supramodal and can operate independently of modality, suggesting a more sensitive state of readiness for multiple inputs to deal with the upcoming challenges in a game. Donohue et al. (2010) also reported that AVGPs can distinguish visual and auditory stimuli that occurs at the same moment or are slightly offset in time, revealing an enhanced alerting ability in both modalities. Since the enemies in AVGs are often recognized by motion or luminance transients, the alerting function influenced by playing AVGs may reveal the plastic bottom-up attention mechanism.

There were more correlations among RTs for the three attentional effects in the AVGPs than in the NAVGPs in the auditory task, suggesting that AVGs are associated with better attentional skills (Green and Bavelier, 2003). The correlation between alerting and conflict was stronger for AVGPs, while the correlations for other functions were tighter for NAVGPs in the visual task, suggesting that AVGs benefit abilities associated with alerting and executive control. AVG playing has been found to be associated with neural changes in the prefrontal cortex (Moisala et al., 2017), left dorsolateral prefrontal cortex and frontal eye fields (Kuhn et al., 2014). This suggests the relationship between AVGs and executive control, which refers to the higher-level ability to focus on the task and ignore noise over space and time (Green and Bavelier, 2015). Notably, the results of the present study showed that AVGPs not only heightened correlations for the executive control effect, which is likely associated with top-down processes (Fan, 2014), but also facilitated more connections with the alerting that exists as an exogenous form of attention, which is considered to be mediated by bottom-up processes (Fan et al., 2002). The results indicate the effects of AVGs on both top-down and bottom-up processing of attention.

There were some limitations to the present study. First, the impact of AVGs on the attentional functions suggests directionality. As the attentional functions of participants before they started to play AVG are unclear, the possibility of attentional functions having an effect on AVG playing cannot be completely ruled out. Future research can utilize a training design to explore the directionality issue (Green and Bavelier, 2007, 2015; Bavelier and Green, 2019). In the training design, participants are randomly assigned to play AVGs and the impact of AVGs can be assessed by comparing the cognitive measures taken before and after training. Second, although Feng et al. (2007) found that playing AVGs can reduce gender differences in spatial attention and spatial cognition, the effect of AVGs on different attentional functions remains unclear. To control for the possible effect of imbalanced gender ratio between groups, we included gender as a covariate in our analyses (i.e., ANCOVA), and the effects of demographic variables in groups differing in gaming experience should be further investigated.

## Conclusion

The results of the present study confirmed the benefits of AVGs for attentional functions, especially for the interaction between orienting and executive control, and the supramodal alerting function. Complementing research on the benefits of AVGs in other domains, such as motivation (Ventura et al., 2013), emotion (Aldao et al., 2010) and social behavior (Gill, 2012), the findings of the present study provide insight into the potential positive implications of video games. As some studies have investigated the effects of various types of video games (Dobrowolski et al., 2015) and the neural mechanisms underlying different attentional functions in ordinary people (Liu et al., 2010), future research employing brain imaging techniques is required to explore the different effects of varied games on the neural mechanisms of attentional functions.

## Data Availability Statement

The raw data supporting the conclusions of this article will be made available by the authors, without undue reservation.

## Ethics Statement

The studies involving human participants were reviewed and approved by the Ethics Committee of Tianjin Normal University. The patients/participants provided their written informed consent to participate in this study.

## Author Contributions

XW and YiJ conceived the study. YiJ analyzed the data. All authors discussed the results and contributed to the writing of the manuscript.

## Conflict of Interest

The authors declare that the research was conducted in the absence of any commercial or financial relationships that could be construed as a potential conflict of interest.

## Footnotes

1 https://www.psychometrica.de/correlation.html

2 The effects were calculated by subtraction, so that they reflect the relative magnitude of values rather than the absolute values of RTs.
